# Paraoxonase I Activity and Its Relationship with Nutrition in Amyotrophic Lateral Sclerosis

**DOI:** 10.3390/antiox13081021

**Published:** 2024-08-22

**Authors:** Belén Proaño, María Benlloch, Sandra Sancho-Castillo, Jesús Privado, Guillermo Bargues-Navarro, Claudia Emmanuela Sanchis-Sanchis, Palmira Martínez Bolós, Ana Belén Carriquí-Suárez, Laura Cubero-Plazas, Jose Luis Platero Armero, Dolores Escriva, Jose Joaquín Ceron, Asta Tvarijonaviciute, Jose Enrique de la Rubia Ortí

**Affiliations:** 1 Doctoral School, Catholic University of Valencia, 46001 Valencia, Spain; beprool@mail.ucv.es (B.P.); guillermo.bargues@mail.ucv.es (G.B.-N.); claudiaemmanuela.sanchis@mail.ucv.es (C.E.S.-S.); a.carriqui@mail.ucv.es (A.B.C.-S.); 2 Department of Basic Biomedical Sciences, Catholic University of Valencia, 46001 Valencia, Spain; joseenrique.delarubi@ucv.es; 3 Department of Pathology, Catholic University of Valencia, 46001 Valencia, Spain; sandra.sancho@ucv.es; 4 Department of Methodology of Behavioral Sciences, Universidad Complutense de Madrid, 28223 Madrid, Spain; jesus.privado@pdi.ucm.es; 5 Health Research Institute (IIS) La Fe, La Fe Polytechnic and University Hospital, 46026 Valencia, Spain; 6 Department of Nursing, Catholic University of Valencia, 46001 Valencia, Spain; palmiramartinezbolos@gmail.com (P.M.B.); laura.cubero@ucv.es (L.C.-P.); jl.platero@ucv.es (J.L.P.A.); 7 Institute for Research on Musculoskeletal Disorders, Catholic University of Valencia, 46001 Valencia, Spain; dolores.escriva@ucv.es; 8 Intensive Care Unit, La Fe Polytechnic and University Hospital, 46026 Valencia, Spain; 9 Interdisciplinary Laboratory of Clinical Analysis, Campus of Excellence Mare Nostrum, University of Murcia, 30100 Murcia, Spain; jjceron@um.es (J.J.C.); asta@um.es (A.T.)

**Keywords:** paraoxonase-1, nutrition, pulmonary function, high density lipoprotein, ALS

## Abstract

**Background**: Amyotrophic lateral sclerosis (ALS) is characterized by progressive motor neuron degeneration, with oxidative stress playing a key role. Paraoxonase 1 (PON1) is an antioxidant enzyme that may influence ALS progression. This study aimed to establish a predictive model for the influence of PON1 activity on functionality in ALS patients and explore its relationship with nutrition. **Methods:** In this observational cross-sectional study, 70 ALS patients underwent assessments of PON1 activity, lipid profile, functional capacity, respiratory function, and heart rate variability. A structural equation model was developed to determine the relationships between variables. Nutritional intake was analyzed in 65 patients. **Results:** The predictive model showed that PON1 activity and LDL levels positively influenced functionality, both directly and indirectly through respiratory capacity. Heart rate variability moderately predicted functionality independently. HDL levels were not significantly associated with functionality. Weak to moderate correlations were found between PON1 activity and intake of certain nutrients, with positive associations for monounsaturated fats and vitamin D, and negative associations for carbohydrates, proteins, and some micronutrients. **Conclusions:** PON1 activity appears to play an important role in ALS patient functionality, both directly and through effects on respiratory capacity. However, its relationship with nutritional intake was not strongly evident in this sample population.

## 1. Introduction

Amyotrophic lateral sclerosis (ALS), also referred to as Lou Gehrig’s disease, is a progressive condition that leads to the degeneration of motor neurons [1]. The malady is characterized by progressive damage and death of lower motor neurons located within the spinal cord and brainstem, as well as upper motor neurons in the motor cortex [2]. Disease progression tends to be swift, with a typical survival period averaging only 2–3 years from the onset of symptoms, usually as a consequence of respiratory failure [3], which is accompanied by a loss of functional capacity in several domains (bulbar, arm/leg motor, and respiratory) [4]. In spite of extensive investigations, the root cause of the condition remains unknown in most cases, and the mechanisms resulting in motor neuron destruction appear to be intricate and not yet fully understood. Nevertheless, it is known that oxidative stress is a mechanism of neurodegeneration in ALS and that antioxidant enzymes play a role in its development [2,5]. Motor neurons are particularly sensitive to oxidative damage because of their intensive metabolism and low levels of endogenous antioxidants [6]. Furthermore, the accumulation of mutated proteins, such as the antioxidant enzyme superoxide dismutase (SOD1), in cases of familial ALS, generates an increase in reactive oxygen species that damage motor cells [5].

Paraoxonases (PONs) are significant enzymes thought to play a significant role in the development of ALS [7]. PONs are enzymes that modulate inflammation and act as antioxidants. PONs originated from carboxylesterase enzymes but have different gene and protein sequences [8]. The PON family comprises three components, PON1, PON2, and PON3, which have similar activities and 90% structural similarity. While PON1 and PON3 are hepatically synthesized and circulate in serum bound to high density lipoprotein (HDL), PON2 is synthesized locally in different tissues [9].

The antioxidant activity of PON1 prevents LDL and HDL oxidation and promotes antioxidant and antiatherogenic activities that have been attributed to HDL [10]. Shifts in PON1 activity and HDL function have been associated with pathophysiological conditions, such as atherosclerosis or neurodegenerative diseases [11].

PON1 acts against oxidative stress in several ways. It is capable of hydrolyzing lipid hydroperoxides derived from lipid peroxidation, thereby preventing the spread of oxidation in membranes [12].

Therefore, lower levels of this enzyme may lead to increased oxidative damage in ALS. Related to this, the relationship between sporadic ALS and PON1 activity has been investigated by different studies that have shown a possible role of defects in *PON1* gene in the development of ALS [13], having identified up to seven mutations of the *PON* gene that are predicted to alter the function of PON in patients with both familial and sporadic ALS [7].

The activities of PONs have been related not only to their direct antioxidant capacity but also to the improvement in different organic functions. In this sense, PON activity is correlated with lung function in cardiovascular diseases [14] and with respiratory problems, especially when PON1 levels are low [15,16], and alterations are associated with a decrease in heart rate variability [17].

It is because of all this evidence that the importance of PON1 in ALS seems clear, as it has been observed that the activity of this enzyme depends to a large extent on nutritional intake [18,19]. Although the relationship between malnutrition and poor prognosis is widely known for ALS [20], the macronutrients most related to PON1 activity in this pathology have not yet been identified.

Because of all these aspects, the objective of this study was to establish a predictive model that provides information on the influence of PON1 enzyme activity on functionality. For this, its role is analyzed individually or through HDL and LDL, and also by taking into account its activity through respiratory capacity or heart rate variability.

## 2. Materials and Methods

### 2.1. Study Design and Population

An observational, cross-sectional study was conducted with a sample of ALS patients with both bulbar and spinal onsets from different locations of Spain.

The sample of the population was collected from the major ALS associations across the state, which were informed about the study beforehand. Participants interested in the study were provided with information regarding the study, including the specified objectives, the tests and analyses to be conducted, and were required to sign an informed consent form. Participants were then assessed against a series of selection criteria. The inclusion criteria were males over 18 years of age, females over 50 who were not fertile or between 18 and 50 years old; patients diagnosed with ALS at least six months prior to the study; patients receiving riluzole treatment; and those who agreed to participate by signing the informed consent form. The exclusion criteria included patients with a tracheostomy; patients with invasive or non-invasive ventilation with positive ventilatory pressure; those involved in another trial within 4 weeks prior to inclusion; patients with evidence of dementia; those dependent on alcohol or drugs; patients infected with hepatitis B or C or who were human immunodeficiency virus-positive; patients with renal issues indicated by creatinine levels twice the normal limit 30 days before inclusion; and patients with liver issues shown by ALT or AST levels three times the normal limit 30 days before inclusion.

After applying these criteria, the definitive population sample to develop the predictive model was 70 ALS patients (with a mean age of 56.19 years ± 10.01 years), with 60% being males, and with 18.6% bulbar ALS and 81.4% spinal ALS. The time from diagnosis was 27.81 ± 27.83 months.

Of this population, nutritional intake determination was performed in 65 patients, with a mean age of 56.48 ± 10.03 years, with 61.5% being males, and whose percentages of bulbar and spinal ALS were 20% and 80%, respectively. The time from diagnosis was 28.12 ± 28.56 months.

### 2.2. Serum Extraction Procedure

Blood samples were obtained from 70 patients diagnosed with ALS following a standardized protocol. Briefly, blood tests were carried out in the peripheral vein (antecubital vein) at 11 a.m. on an empty stomach. The blood samples were collected in BD Vacutainer Plus serum blood collection tubes. After that, blood samples were centrifuged to separate serum at 1500× *g* 15 min (Thermo Scientific Sorvall ST, San Diego, CA, USA, 40R centrifuge). Serum samples were frozen at −80 °C until they were analyzed.

### 2.3. Determination of the Lipid Profile and PON 1 Activity

Lipid analytes (HDL and LDL) were calculated by commercially available assays (Beckman Coulter) in an automated biochemistry analyzer (Olympus AU400, Beckman Olympus AU400 Chemistry Analyzer, Beckman Coulter, Brea, CA, USA).

PON1 activity was measured using 4-nitrophenyl acetate (Sigma-Aldrich, Louis, MI, USA) by a previously described assay [21]. Measurements were made using an automated clinical biochemistry analyzer (Olympus AU400, Chemistry Analyzer, Beckman Coulter, Brea, CA, USA).

### 2.4. Measure of Functional Capacity

For the 70 patients, functional ability was also measured with the revised ALS Functional Rating Scale (ALSFRS-R test). This is a sensitive, accurate, and reproducible scale that assesses functional capacity considering the domains of deterioration: bulbar, upper limb, lower limb, and respiratory. The ALSFRS-R test consists of 12 items grouped into four fields that graduate disabilities in activities of daily living. Each function is scored from 4 (normal) to 0 (no ability). Therefore, the rank of total functional disability thus varies from 0 (maximum disability) to 48 (normal) points [22]. 

### 2.5. Assessment of Respiratory Capacity: Spirometry Forced Expiratory Volume in 1 S (FEV1)

There are different methods of assessing respiratory function in ALS, among which spirometry distinguishes [23], which allows for the measurement of the forced expiratory volume in 1 s (FEV1), among other determinations [24]. A MasterScreen PFT powered by SentrySuite™ (Jaeger, Alemania) was used for its determination and was performed by a nurse in accordance with the guidelines of the Spanish Society of Family and Community Medicine [25]. Prior to starting the procedure, calibration/verification of the spirometer was performed following the protocols established for this purpose. Three acceptable and repeatable tests were performed [26], and the best test was selected. 

### 2.6. Heart Rate Variability (HRV)

HRV measures the variability in time between RR intervals on an electrocardiogram, reflecting the physiological differences in the intervals between heartbeats [27]. HRV analyses were used, and the Elite HRV application collected heart rate data from the Polar H7 Bluetooth sensor, with heart rate sampling frequencies adjustable to 1 Hz, 2 Hz, 4 Hz, or 5 Hz. Data underwent an initial process of cleaning to eliminate artifacts and noise, allowing for a maximum of 5% interference, as these can significantly affect the results [28]. Heart rate intervals that are excessively short or long are then excluded, and various HRV metrics are computed. The HRV data can be displayed as graphs and trends, and personalized reports can be generated to interpret the data.

### 2.7. Nutritional Analysis

Sixty-five patients also underwent nutritional analysis, for which the following measurements were performed.

#### 2.7.1. Dietary-Nutritional Anamnesis and Collection of Data

For the initial analysis of the patients’ intake, 24 h records over a period of 7 days [29] and the Food Frequency Questionnaire [30] were used. Both records provided information on the frequency of the consumption of different food groups, including dairy products, vegetables, fruits, juices, nuts, meat, fish, seafood, eggs, tubers, rice, legumes, pasta, sausages, snacks, pastries, cookies, chocolates, soft drinks, fermented alcohol, and distilled alcohol. This 7-day period allowed for sufficient information to be collected about the patients’ usual diet, minimizing the risk of bias associated with the choice of one day per week [29]. The 24 h record is a self-completion questionnaire [29] in which the patient must record the type of food consumed daily, as well as the different ingredients used to prepare each dish, indicating the homemade measure (a cup, a serving, a glass, a tablespoon, a slice, a handful, a plate, a ladle) or the exact weight of the food or beverage. To facilitate the task for study participants, information on the weight of each serving and the most common measurement units was provided [31]. Furthermore, using the Food Frequency Questionnaire, participants completed a table in which they were asked how often they typically consumed each food group.

#### 2.7.2. Assessment of Diet and Eating Habits

Considering the patients’ daily dietary record over 7 days and the Food Frequency Questionnaire, the quality of the diet was calibrated using DietoPro^®^ software https://dietopro.com/ (Dietary-nutritional management software, Valencia, Spain). Using this program, a nutritional profile was obtained using a daily average calculation of macro- and micronutrients. The software calculated daily macronutrient levels: energy intake, total proteins, total carbohydrates, total lipids, lipid profile (monounsaturated, saturated, and polyunsaturated fatty acids), cholesterol, and the percentage distribution of macronutrients (proteins, lipids, and carbohydrates) from the meals inputted into the program by nutritionists. Regarding the micronutrients, the software daily calculated the amount of sodium, fiber, ethanol, iodine, potassium, calcium, magnesium, phosphorus, iron, selenium, zinc, vitamin B1, vitamin B2, vitamin B6, vitamin B12, folate, niacin, vitamin C, vitamin A, vitamin D, and vitamin E, as well as the balance of calcium/phosphorus, vitamin E/polyunsaturated fatty acids, and vitamin B6/protein. Furthermore, to assess whether the participants’ diets were adequate, “Dietary Reference Intake” (DRI) from the «Spanish population’s reference intakes» [32] and the «Consensus of the Spanish Society of Community Nutrition (SENC)» [33] was used as a guide.

### 2.8. Statistical Analysis

First, the distribution of the different measures collected was calculated to see if they presented a normal distribution and the descriptive statistics of the same, using the SPSS statistical package V 21.

Pearson’s correlations were calculated between the different variables that were going to be part of the predictive model to see the possible tendency of association between them.

Third, a structural equation model, specifically a path analysis, was performed using the AMOS V. 23 program [34] to determine the predictive role of PON1 and lipoproteins (HDL and LDL) on spirometry (FEV1), HRV, and the functional capacity of ALS patients. Spirometry and heart rate variability were considered mediating variables between PON1 and lipoproteins on functional capacity. To evaluate the data’s alignment with the tested model, two types of goodness-of-fit indices were utilized: (1) Absolute indices to determine if the theoretical model aligns with the empirical data collected. These included the χ2/df index [35], with values below 3 indicating a good fit; the Goodness-of-Fit Index (GFI) [36], where values above 0.95 are considered a good fit; the Standardized Root Mean Square (SRMR) [37]; and Root Mean Squared Errors (RMSEs) [38], with values less than 0.08 indicating a good fit [39], and having less than 5% of standardized residuals greater than 2.58 in absolute value [36,39]. (2) Incremental indices were used to compare the obtained model with the null model. These included the Normed Fit Index (NFI) [35] and the Comparative Fit Index (CFI) [40], which had values above 0.95 indicating a good fit. For this type of model, it is generally recommended to have 10 participants per indicator or variable [41], although others suggest that 5 per indicator is sufficient when the distribution is normal [38]. In our case, we meet the second criterion by having 70 participants for six indicators (70/6 = 11.67).

Finally, Pearson’s correlation was calculated between PON1 and different nutritional measures to determine the degree of association.

### 2.9. Ethical Concerns

This study was conducted in accordance with the Declaration of Helsinki [42]; prior approval of the protocol was obtained by the Institutional Ethics Committee of Clinical Research at Hospital La Fe in Valencia, Spain (protocol code 2021-001989-38).

## 3. Results

### 3.1. Descriptive Analysis

Table 1 shows the descriptive statistics and distribution of the various measures taken in ALS patients that will be used for the predictive model (HDL, LDL, PON1, FEV1, HRV, and functionality) and the correlations of PON1 with nutritional values. Regarding the measures used in the predictive model, all showed appropriate values of skewness (not exceeding 2 in absolute value) and kurtosis (not exceeding 7 in absolute value), indicating that they would exhibit a normal distribution following the values recommended by West et al., 1995 [43], for models of this type, estimated using the maximum likelihood procedure. However, for different nutritional values, numerous cases do not present a normal distribution. Therefore, the results should be interpreted with caution when calculating the Pearson correlation between these measures and PON1.

### 3.2. Correlations between PON1 Activity and the Other Variables in ALS

The correlations between the variables are presented in Table 2. According to Cohen [44], the effect size of the correlation was considered low for values below 0.10, moderate up to 0.30, and high from 0.60 onwards. The effect size indicates the importance of the correlation at a population level; the larger the effect size, the more significant the obtained result is at a population level, and therefore, the more generalizable the results are. There were three correlations with a moderate effect size: spirometry (FEV1) with HDL (r = 0.30) and LDL (r = 0.34) lipoproteins and FEV1 with functionality (r = 0.34), and three correlations with a low to moderate effect size: FEV1 with PON1 (r = 0.21), HRV with functionality (r = 0.22), and PON1 with functionality (r = 0.21). These results indicate that PON1 has relationships with certain effect sizes with the rest of the measures, which would justify proposing a predictive model to explain the functionality of ALS patients.

### 3.3. PON1 Activity in ALS: Predictive Model

**Figure 1:**
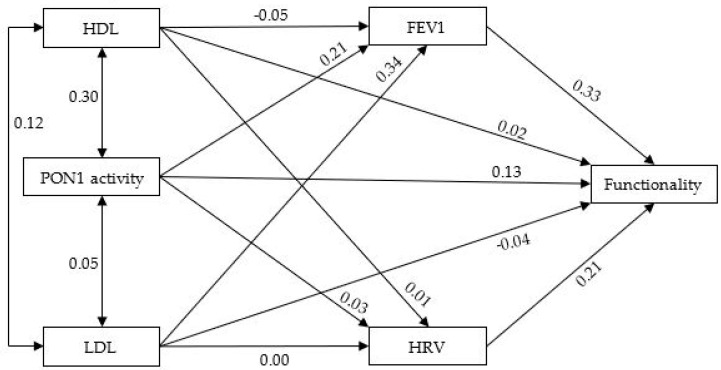
Predictive model of PON1 activity, lipoproteins (HDL and LDL), spirometry (FEV1), HRV, and functionality in ALS.

The contrast predictive model is shown in Figure 1. The adjustment of the model to the data was very good (χ2/df = 0.01, GFI = 1.000, NFI = 1.000, CFI = 1.000, RMSEA = 0.000, SRMR = 0.002, and no residual values exceeded 2.58); therefore, we have high confidence that the theoretical model fits the empirical data. In the model, we assumed that the predictors were PON1 and lipoproteins (HDL and LDL), the mediating variables were spirometry (FEV1) and heart rate variability (HRV), and the criterion was the functionality of ALS patients.

Regarding the predictors, only PON1 and HDL showed a relationship with a medium effect size, according to Cohen [44] (r = 0.30), indicating a certain positive association between both variables, meaning that higher PON1 scores correspond to higher HDL values. 

The remaining values in the model are the weights of the linear regression. According to Cohen [44], an f^2^ value of R^2^/(1 − R^2^) of 0.02 is a small effect size, 0.15 is medium, and 0.35 is large. By rearranging the formula, we find that an r value of 0.14 is a small effect size, 0.36 is medium, and 0.51 is large. We can observe that the respiratory function determined by spirometry (FEV1) is positively explained by PON1 activity (β = 0.21) and LDL (β = 0.34), with both variables accounting for 16% (R^2^ = 0.16) of spirometry variance. In other words, higher PON1 and LDL scores correlated with higher spirometry. With regard to HRV, none of the measures can adequately explain it, as their β values are below 0.14. Functionality was directly explained by 18% (R^2^ = 0.18) of the variance in PON1 (β = 0.13), HRV (β = 0.21), and FEV1 (β = 0.33). Additionally, PON1 indirectly explained functionality by positively predicting FEV1 (β = 0.21), which in turn affected functionality (β = 0.33). Thus, PON1 has a dual effect on functionality, one direct and one indirect, through spirometry. Similarly, LDL has an indirect effect on functionality through spirometry.

### 3.4. Relation between Nutrition and PON1 Activity

Table 3 presents the correlations between PON1 and the macro- and micronutrients in patients with ALS. Considering values with a small effect size according to Cohen’s criterion [44], that is, at least 0.10, PON1 was positively associated with MUFA (r = 0.21) and vitamin D (r = 0.22), leading to an increase in its activity with these nutrients in ALS. PON1 exhibits negative relationships, decreasing its values with carbohydrates, protein, sodium, fiber, iodine, potassium, phosphorus, selenium, vitamins, phosphate, and vitamins A and C. Moreover, when considering a medium effect size (0.30), PON1 was negatively correlated with magnesium (r = −0.31). Hence, high magnesium levels reduce PON1 activity in ALS patients.

## 4. Discussion

Oxidative stress in the CNS plays a central role in the pathology of ALS [45]. Thus, PON1 could be an upward marker for determining the oxidation state of ALS. The importance of the association between PON1 polymorphisms and the development of ALS has already been observed in the context of a high incidence of sporadic ALS among young veterans of the Gulf War who had been exposed to organophosphate pesticides [46]. As explained in the Introduction section, defects in the *PON1* gene play a role in ALS development. To further understand the possible relationship between PON1 activity and ALS, we developed a predictive model in the present work, where we effectively evaluated the predictive role of PON1 and the functional capacity of these patients. ALSFRS-R is commonly used to determine the progression of the pathology, and in our model, it was correlated with PON1 activity. Thus, patients who show the highest levels of PON1 enzyme activity are also those with the greatest functional capacity. Additionally, it is worth highlighting that this relationship occurs not only directly but also through a relation of PON1 with respiratory capacity (determined by the FEV1 spirometry test), coinciding with a previous study that already showed respiratory difficulties associated with greater oxidative stress determined by decreased PON1 levels [15]. Therefore, according to our results, functional capacity in ALS can be directly and indirectly explained through PON1. In this sense, it is worth highlighting that PON1 and HDL showed a relationship with a medium effect size (Figure 1), as expected [47]. Part of PON1 is bound to HDL and is involved in the antioxidant activity of this lipoprotein [10]; nevertheless, PON1’s role on functionality does not appear to depend on the concentration of this lipoprotein, since HDL does not predict respiratory capacity or directly predict functionality. In this sense, it has been seen that even high HDL levels could be related to worse survival [48], specifically with lower respiratory capacity [49], which would explain in part why HDL and PON1’s positive activity are not related in ALS patients in the spirometry test. Additionally, it has already been indicated by other authors that the presence and expression of PON1 in lung tissue are independent of HDL [50], which would ultimately justify our results.

Another relevant aspect of our model was the role of LDL. This lipoprotein also seems to explain the positive functional capacity; therefore, high levels predict greater functionality. However, it is worth highlighting that unlike what is observed with PON1 activity, this relationship only occurs indirectly through respiratory capacity. Therefore, high LDL levels could be positive for respiratory capacity as a mediating variable in turn for functional capacity. In this way, it was confirmed that high LDL levels (associated with low HDL) could be positive for these patients, unlike what is observed in most diseases (especially cardiovascular diseases) and coinciding with previous results [51].

Finally, it is also worth highlighting how HRV moderately predicts ALS patient functionality (r = 0.21) (measured by the ALSFRS-R test) independently of PON1 activity and the rest of the variables analyzed (Figure 1). This finding implies that higher HRV is associated with increased functionality in ALS, according to another pilot study that presented some limitations [52].

Given the importance of PON1 in functional capacity, its increase in patients seems especially relevant. Therefore, a relationship between PON1 activity and diet has been observed. Different previous works clearly link both variables [53,54,55], and in the case of ALS patients, this relationship could help understand PON1’s role since there is also clear evidence of the relationship between diet and the disease; it is widely accepted that dietary supplements represent a new approach to improve the health of the ALS population [56], and that early and systematic nutritional intervention on the clinical conditions of ALS patients decreases the percentage of mortality that occurs within the first year of diagnosis [57]. However, in our study, we did not observe an important relationship between both variables, identifying only low or medium relationships, which is in line with what was observed in our laboratory in patients [58]. Specifically, it was observed that with a low correlation, the intake of monounsaturated fatty acids (MUFAs) and vitamin D increased its activity. Regarding MUFAs, it has been seen how oleic acid (omega-9 monounsaturated fatty acid) increases PON1 activity [59], which seems to coincide with our results; in relation to vitamin D (specifically vitamin D3 and/or its metabolites), despite its antioxidant and anti-inflammatory activity [60], and that it directly increases the activity of the antioxidant enzyme in ALS, increasing the protection of motor neurons and neuronal vulnerability to glutamate excitotoxicity [61,62], it has not been described in other works that its intake is related to PON1 activity.

Regarding the impact of protein intake, there is little evidence identifying only one study in which the decrease in PON1 activity (specifically in rats induced by arthritis) was restored after treatment with soy protein and isoflavones (genistein and daidzein) [63]. However, in our study, a negative relationship between protein intake and enzyme activity was found, although these results are not comparable because the relationship occurred with the total intake of protein as a macronutrient and not with specific proteins. On the contrary, the negative relationship with carbohydrate consumption that we observed coincides with studies linking increases in blood carbohydrates in patients with type 2 diabetes with decreased PON1 [64,65].

We also observed a medium negative correlation of PON1 with magnesium and a low negative correlation with intake of sodium, iodine, potassium, selenium, vitamin A, and vitamin B, which has not been previously described. The negative relationship found in our report between vitamin C intake and PON1 seems more surprising since different studies, with the exception of Ardalic et al. (2014), which did not find a relationship [66], agree that the intake of complexes containing this vitamin can lead to an increase in PON1 activity [67,68].

## 5. Conclusions

It appears that PON1, LDL, and respiratory capacity are the most relevant measures used to explain the functionality of ALS patients, with respiratory function moderating the effects of PON1 and LDL on functionality. To a lesser extent, heart rate variability also predicts functionality. The data suggest that HDL does not play a significant predictive role in functionality or any other measures considered in the model, although it is related to PON1. Finally, PON1 activity does not have a strongly significant relationship with the intake of any macro- or micronutrients, only weak correlations with carbohydrates, protein, magnesium, sodium, fiber, iodine, potassium, phosphorus, selenium, vitamins, phosphate, and vitamins A and C.

Despite these conclusions, our study has several limitations. Among these, it is worth noting that a small population sample was used, which could have especially influenced the poor relationship evidenced between the intake of micro- and macronutrients and PON1 activity. In addition, it would be interesting to further deepen the relationship between HDL and PON1 activity, since HDL particles can be divided into large HDL2 subfractions (HDL2a and HDL2b) and small HDL3 subfractions (HDL3a, HDL3b, and HDL3c), which differ in size, structure, and function. Finally, we also propose to evaluate the role of sex in the association between the variables.

Finally, it is worth noting that our study also presents the limitation of not having taken into account the possible existence of polymorphisms in the *PON1* gene. In this regard, it is noteworthy that to date, clear evidence of these polymorphisms in patients or animal models of ALS has not been described in previous studies; however, given the presumed importance of this enzyme activity in the course of the disease, further investigation into this matter is of utmost interest in future studies.

## Figures and Tables

**Table 1:** Descriptive statistics and distribution of the analyzed variables.

Variables	N	Mean	SD	Asymmetry	Kurtosis
HDL	70	51.80	12.42	0.96	0.96
LDL	70	140.78	37.60	1.04	1.85
PON1	70	3.12	0.86	0.65	0.36
FEV1	70	1.77	0.83	0.56	0.30
HRV	70	42.30	11.33	0.12	1.13
Functionality (ALSFRS-R)	70	28.57	8.92	−0.19	−0.40
Energy (kcal)	65	2294.72	853.30	1.39	3.85
Total Carbohydrates (g)	65	222.27	93.52	0.82	0.62
Total Protein (g)	65	115.96	41.30	0.57	0.42
Total fat (g)	65	136.01	136.23	2.89	8.22
SAFA (g)	65	31.22	17.27	1.20	2.34
PUFA (g)	65	39.06	160.03	7.95	63.76
MUFA (g)	65	29.23	27.66	0.73	−0.15
PUFA/SAFAs	65	9.83	32.49	5.95	39.04
(PUFA + MUFA)/SAFA	65	62.91	378.77	8.00	64.26
Cholesterol (g)	65	459.72	231.62	0.36	0.34
Sodium (g)	65	3661.49	10,567.02	7.88	63.02
Fiber (g)	65	30.01	51.56	6.88	51.33
Ethanol (g)	65	86.02	602.09	7.98	64.03
Iodine (µg)	65	226.01	249.30	3.24	11.70
Potassium (mg)	65	3983.11	2287.92	1.83	4.62
Calcium (mg)	65	1022.08	520.22	0.87	0.19
Magnesium (mg)	65	396.29	218.68	1.36	2.71
Phosphorus (mg)	65	1514.69	725.64	1.13	3.09
Iron (mg)	65	33.29	82.87	5.40	28.69
Selenium (µg)	65	126.44	102.57	4.64	29.48
Zinc (mg)	65	18.49	48.19	7.79	61.96
Vitamin B1 (mg)	65	7.67	46.82	8.05	64.83
Vitamin B2 (mg)	65	8.70	47.03	7.93	63.46
Vitamin B6 (mg)	65	13.60	62.32	5.59	30.34
Vitamin B12 (µg)	65	15.58	53.13	7.80	62.09
Folate (µg)	65	325.63	163.30	1.28	2.39
Vitamin B3 (mg)	65	73.92	269.52	7.64	59.93
Vitamin C (mg)	65	175.09	113.99	1.34	2.51
Vitamin A (µg)	65	1127.22	989.45	2.41	6.62
Vitamin D (µg)	65	34.65	166.09	7.27	54.98
Vitamin E (mg)	65	20.56	48.53	7.59	59.76
Calcium/Phosphorus	65	7.3	12.79	4.19	23.77
Vitamin E/PUFA	65	9.16	21.64	3.93	17.77
Vitamin B6/Protein	65	5.37	39.8	8.06	64.97

**Table 2:** Pearson correlations among PON1, lipoproteins (HDL and LDL), spirometry (FEV1), heart rate variability (HRV), and functionality in ALS.

	HDL	LDL	PON1	FEV1	HRV	Functionality
HDL	1.00					
LDL	0.12	1.00				
PON1	0.30	0.05	1.00			
FEV1	0.05	0.34	0.21	1.00		
HRV	0.01	0.01	0.03	0.02	1.00	
Functionality (ALSFRS-R)	0.08	0.08	0.21	0.34	0.22	1.00

Note: Correlations ≥ |±0.30| are statistically significant at 5% level.

**Table 3:** Pearson correlations between PON1 and macro- and micronutrients.

Variables	PON1	Variables	PON1
Energy (kcal)	−0.09	Phosphorus (mg)	−0.16
Total Carbohydrates (g)	−0.21	Iron (mg)	−0.06
Total Protein (g)	−0.22	Selenium (µg)	−0.18
Total fat (g)	0.08	Zinc (mg)	−0.21
SAFA (g)	0.04	Vitamin B1 (mg)	−0.20
PUFA (g)	0.03	Vitamin B2 (mg)	−0.21
MUFA (g)	0.21	Vitamin B6 (mg)	−0.17
PUFA/SAFA	0.01	Vitamin B12 (µg)	−0.20
(PUFA + MUFA)/SAFA	−0.03	Folate (µg)	−0.19
Cholesterol (g)	0.02	Vitamin B3 (mg)	−0.07
Sodium (g)	−0.16	Vitamin C (mg)	−0.15
Fiber (g)	−0.23	Vitamin A (µg)	−0.11
Ethanol (g)	−0.05	Vitamin D (µg)	0.22
Iodine (µg)	−0.20	Vitamin E (mg)	−0.20
Potassium (mg)	−0.10	Calcium/Phosphorus	0.08
Calcium (mg)	−0.03	Vitamin E/PUFA	0.00
Magnesium (mg)	−0.31	Vitamin B6/Protein	0.01

Note: Correlations ≥ |±0.24| are statistically significant at 5% level.

## Data Availability

The data that support the findings of this study are available on request from the corresponding author. The data are not publicly available due to privacy or ethical restrictions.
